# Esketamine hydrochloride in the management of moderate‐to‐severe depressive symptoms in patients undergoing multiple wound repair surgeries: A multi‐centre randomized, double‐blind, placebo‐controlled trial

**DOI:** 10.1002/ctm2.70641

**Published:** 2026-03-20

**Authors:** Xiaomeng Yu, Tianqi Shen, Ting Zhang, Rui Bao, Ziyi Guo, Zirui Feng, Li Tong, Xiaoying Zhang, Mingzi Ran, Guanyong Sun, Weidong Mi, Jingsheng Lou, Qiang Fu

**Affiliations:** ^1^ Department of Anesthesiology The First Medical Center of Chinese PLA General Hospital Beijing China; ^2^ Chinese PLA Medical School Beijing China; ^3^ Department of Anesthesiology No. 984 Hospital of the PLA Beijing China; ^4^ Combat Trauma Center No. 984 Hospital of the PLA Beijing China; ^5^ Department of Anesthesiology The Fourth Medical Center of Chinese PLA General Hospital Beijing China; ^6^ Department of Psychiatry No. 984 Hospital of the PLA Beijing China

**Keywords:** esketamine, Montgomery‐Åsberg depression rating scale, multiple trauma, postoperative complications, postoperative depression, Esketamine, Postoperative Depression, Multiple Trauma, Postoperative Complications, Montgomery‐Asberg Depression Rating Scale

## Abstract

**Introduction:**

Patients undergoing multiple wound repair surgeries often develop moderate‐to‐severe anxiety and depression. However, there is a lack of effective rapid emotional intervention strategies during the perioperative period.

**Methods:**

This multi‐centre, randomized, double‐blind, placebo‐controlled trial involved 130 adult patients (65 in the esketamine group and 65 in the placebo group). Participants were randomly assigned to receive either esketamine (0.2–0.3 mg/kg) or saline intravenously during surgery. The primary outcome was the response rate (proportion of patients with ≥50% reduction in MADRS total score from baseline) on postoperative days (PODs) 1–3, evaluated using the Montgomery–Åsberg depression rating scale (MADRS). The secondary outcome was the remission rate (proportion of patients with MADRS total score ≤10) on postoperative days (PODs) 1–3; scores on the Patient Health Questionnaire‐9 (PHQ‐9), the Hospital Anxiety and Depression Scale‐Anxiety subscale (HADS‐A); and esketamine‐related neuropsychiatric adverse events assessed using the Young Mania Rating Scale (YMRS), Clinician‐Administered Dissociative States Scale (CADSS), and Brief Psychiatric Rating Scale (BPRS) within 30 days after surgery.

**Results:**

The esketamine group showed a significantly higher response rate than the placebo group on POD 1–3. (POD 1: 53.8% vs. 26.2%, *p *= 0.001; POD 2: 60.0% vs. 40.0%, *p *= 0.009; POD 3: 73.8% vs. 53.8%, *p *= 0.018). The esketamine group also showed a higher remission rate and lower MADRS scores (POD 1: 33.8% vs. 10.8%, *p *= 0.002; POD 2: 40.0% vs. 23.1%, *p *= 0.038; POD 3: 56.9% vs. 23.1%, *p *< 0.001). Esketamine improved HADS‐A and PHQ‐9 scores by POD 3 without increasing neuropsychiatric adverse events within 30 days postoperatively.

**Conclusions:**

The results demonstrate that the intraoperative use of low‐dose esketamine can rapidly and effectively alleviate moderate‐to‐severe anxiety and depressive symptoms in the early postoperative period (POD 1–3) among patients requiring repeated debridement surgeries without increasing neuropsychiatric or systemic adverse events within 30 days after surgery.

## Highlights

- The esketamine group demonstrated significantly higher antidepressant response rates compared with the placebo group at postoperative day 1 (53.8% vs. 26.2%), day 2 (60.0% vs. 40.0%), and day 3 (73.8% vs. 53.8%)
- The esketamine group exhibited markedly higher depression remission rates and significantly lower scores on the Montgomery‐Åsberg Depression Rating Scale (MADRS)
- No statistically significant increase in psychiatry‐related adverse events was observed in the esketamine group relative to the control group across follow‐up periods (postoperative days 13, week 2, and month 1).

## Graphical abstract

1. Multi‐centre, double‐blind, randomized controlled trial.

2. No esketamine‐related adverse events within 30 days postoperatively.

3. Esketamine group showed higher MADRS response and remission rates.

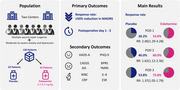

## 1 INTRODUCTION

Depression and anxiety are common mental health issues among patients during the perioperative period, and severe depression increases the likelihood of suicidal behaviour.^1^ Studies have indicated that patients who require repeated surgical interventions often experience moderate‐to‐severe anxiety and depression during the perioperative period.^2^, ^3^ For this patient population, the impact of an elevated risk of adverse perioperative outcomes due to emotional issues^4^ includes increased consumption of opioids immediately after surgery, a higher probability of readmission, delirium, elevated incidence of postoperative cardiac events, and reduced quality of life at 1 year after surgery.^4^, ^5^, ^6^, ^7^, ^8^, ^9^ Accordingly, patients who require repeated surgical debridement and repair are more likely to develop symptoms of moderate‐to‐severe anxiety and depression, with perioperative acute anxiety and depression are most pronounced during the first 24 to 72 h following surgery.^10^

According to previous studies,^11^, ^12^ esketamine can ameliorate anxiety and depression in patients during the perioperative period of various surgical interventions^13^, ^14^ and improve sleep quality,^15^ while its use is associated with mild adverse effects.^13^ Nonetheless, these studies have not sufficiently differentiated the severity of preoperative anxiety from that of depression. Considering the impact of the severity of emotional states on adverse perioperative outcomes, patients with perioperative moderate‐to‐severe anxiety and depression require more care and timely interventions to alleviate their perioperative emotional states than patients with mild anxiety and depression. However, the effectiveness and safety of intravenous esketamine administration during the perioperative period remain uncertain in patients with perioperative moderate‐to‐severe anxiety and depression.^16^

There is a lack of high‐quality randomized controlled trial (RCT) evidence targeting high‐risk patients with moderate‐to‐severe emotional disorders who require repeated surgeries within a short period, particularly with regard to rapid perioperative intervention strategies. Most trials examining the impact of esketamine on perioperative anxiety and depression have focused on single‐procedure populations.^15^, ^17^, ^18^ In contrast, we screened patients with scale‐assessed moderate‐to‐severe anxiety and depression who had undergone multiple surgeries, aiming to verify whether esketamine can rapidly alleviate their symptoms of anxiety and depression in the short term and, thereby, improve the prognosis.

According to the Patient, Intervention, Comparison, and Outcome (PICO) framework (PICO) framework, this study aims to investigate, through a randomized, double‐blind, placebo‐controlled trial, whether a single intraoperative subanaesthetic dose of esketamine (0.2–0.3 mg/kg) can evaluate the postoperative neuropsychiatric response and confirm the perioperative safety in patients undergoing repeated wound repair surgeries who present with moderate‐to‐severe anxiety and depression preoperatively. This study hypothesizes that, compared with placebo, the esketamine group will demonstrate a significantly higher emotional response rate within 3 days postoperatively without an additional increase in the incidence of adverse events within 30 days.

## 2 METHODOLOGY

### 2.1 Study design

This prospective, multi‐centre, randomized, placebo‐controlled, double‐blind trial was approved by the Ethics Committee of the People's Liberation Army General Hospital (S2023‐257‐01) and registered with the Chinese Clinical Trials Registry (ChiCTR2300078540). All patients and their legal representatives provided written informed consent. This study was conducted without using artificial intelligence (AI) tools in accordance with the Transparency in the Reporting of Artificial Intelligence (TITAN) guidelines 2025.^19^ The results were reported using the Consolidated Standards of Reporting Trials (CONSORT) statement.^20^

### 2.2 Patients

All patients were from two tertiary hospitals in Beijing, China, affiliated with the Chinese PLA General Hospital. Both centres operated under an identical protocol, a common randomization sequence, and a unified case report form. Research nurses and outcome assessors who had undergone joint training handled participant screening, randomization, and follow‐up to ensure consistency in intervention delivery and outcome assessment. The sample size allocation across centres was based on the anticipated proportion of eligible outpatients that each site could enrol.

The study enrolled adult patients aged 18 years or older, classified as American Society of Anesthesiologists physical status classification system (ASA) grade I–II. Patients were required to have a history of multiple (two or more) trauma repair surgeries and were scheduled to receive general anaesthesia. The study included only patients with moderate‐to‐severe depression, as indicated by a Patient Health Questionnaire 9 (PHQ‐9) score of 10 or higher^21^ and a Montgomery‐Åsberg Depression Rating Scale (MADRS) score of 22 or higher^22^ (inclusion was based on scale scores and that a formal diagnostic interview for MDD was not part of the screening process). The exclusion criteria were as follows: other mental disorders; contraindications for esketamine or a history of opioid allergy; a history of cerebral infarction; recent use of hormonal therapy; chronic alcohol abuse; long‐term use of sedatives or analgesics; diabetes mellitus; autoimmune rheumatic disease; preoperative haemoglobin concentration below 90 g/L; prior diagnosis of depression with pharmacological treatment; anticipated need for postoperative intensive care unit (ICU) admission; a mini‐mental state examination (MMSE) score below 18; and lack of an informed consent form. Additionally, to ensure protocol integrity during trial conduct, two further criteria were applied: (1) inability to complete the comprehensive preoperative psychological assessment (e.g., due to admission timing), and (2) an estimated surgical duration of less than 30 min or more than 7 h. A corresponding protocol amendment to update the trial registry has been submitted. This stringent screening process ensured that the study focused on a specific population with a clearly elevated risk of moderate‐to‐severe depression and anxiety resulting from multiple surgical procedures, while also minimizing potential confounding factors that could affect the research outcomes.

### 2.3 Randomization and blinding

Centre‐stratified block randomization was used, with the centre as the stratification factor (two strata). Within each centre, randomly permuted block sizes of 4 and 6 were applied to generate a 1:1 allocation sequence. The randomization list was pre‐generated by an independent statistician and stored in a password‐protected Excel file accessible only to an independent researcher who was not involved in participant enrolment or outcome assessments. Investigators contacted the researcher for each randomization assignment to ensure allocation concealment.

Esketamine was formulated by researchers not involved in other procedures. An independent researcher prepared the study solution, which was filled into a syringe labelled ‘Study Medication’ (50 mL). The two solutions were indistinguishable in terms of their appearances. The patient, anaesthesiologist and postoperative assessor were blinded to the group allocation.

### 2.4 Drug administration

In this study, after induction and tracheal intubation, patients in the experimental group were immediately administered intravenous esketamine at 0.2–0.3 mg/kg via pump infusion for 20 min. The placebo group received the same volume of normal saline 20 min after intubation. Induction, maintenance and emergence from anaesthesia followed established clinical protocols (see below).

### 2.5 Anaesthesia induction and intraoperative monitoring

After the patient was placed in the supine position, an intravenous line was established using a 20‐G cannula, and balanced fluid was infused at 4 mL/kg/h. An IntelliVue MP50 instrument (Philips) continuously monitored the electrocardiogram (ECG), heart rate (HR), blood pressure (BP), and blood oxygen saturation (SpO_2_) while recording vital signs.

For induction, the patients were pre‐oxygenated with a face mask for 3 min at a flow rate of 6 L/h. Midazolam 0.01–0.02 mg/kg, etomidate 0.05–0.1 mg/kg, propofol 1–1.5 mg/kg, rocuronium 0.6–1 mg/kg, and sufentanil 0.3–0.4 µg/kg were given intravenously. Endotracheal intubation was performed after anaesthesia induction. Mechanical ventilation was set at VT 6–8 mL/kg, RR 12–14 breaths/min, targeting a PETCO_2_ of 35–45 mmHg. Anaesthesia was maintained with sevoflurane 1–2 MAC, propofol 4–8 mg/(kg·h), remifentanil 0.1–0.2 µg/(kg·h), and intermittent rocuronium 10–20 mg.

Standard general anaesthesia monitoring was performed, including ECG, HR, BP, end‐tidal carbon dioxide (ETCO_2_), SpO_2_, nasopharyngeal temperature, and bispectral index (BIS).

### 2.6 Anaesthesia management and intervention measures

The depth of anaesthesia was adjusted based on the BP, HR and BIS values. The BIS was maintained at 40–60. For systolic blood pressure (SBP) changes exceeding 20% from the baseline, urapidil (0.2–0.5 mg/kg) or ephedrine (0.1–0.3 mg/kg) was administered. For HR above 110 beats/min, esmolol (0.5–1.0 mg/kg) was administered, and for HR below 50 beats/min, atropine (0.01 mg/kg) was used. Active cutaneous warming was applied to patients with a body temperature below 36.0°C to maintain their body temperature above this level. Thirty minutes before the end of surgery (if the duration exceeded 2 h), sufentanil 5–10 µg was readministered. Postoperatively, patients received flurbiprofen ester 50 mg and tropisetron 5 mg for multimodal analgesia and antiemesis. The analgesia regimen comprised sufentanil 2–2.5 µg/kg + tropisetron 0.02 mg/kg + saline to 80 mL, infused at 1 mL/h, with a bolus volume of 0.5 mL/15 min. The patients were returned to the ward after extubation and complete anaesthesia.

### 2.7 Primary outcome

The primary outcome metric in this investigation was the response rate, defined as the proportion of patients manifesting a ≥50% decrease in the MADRS score from baseline on postoperative days (POD) 1–3.^23^, ^24^ MADRS is a widely recognized tool for assessing the severity of depressive symptoms and provides a quantitative measure that helps evaluate treatment outcomes. A ≥50% reduction in the MADRS score is widely recognized as a significant indicator of treatment response, reflecting a marked reaction to treatment.^25^, ^26^ Using this metric, we aimed to evaluate the effectiveness of esketamine in alleviating postoperative depressive symptoms and effectively capture the extent of symptom improvement in patients who received treatment.^27^, ^28^

### 2.8 Secondary outcomes

The secondary outcome metrics encompassed remission rates evaluated on POD 1–3, defined as a post‐treatment MADRS score ≤10, consistent with previous research on the impact of esketamine on depressive symptoms in patients.^28^ The MADRS was used as a continuous variable to assess symptom severity. On POD 3, anxiety severity was assessed using the Hospital Anxiety and Depression Scale‐Anxiety scale (HADS‐A), with a score ≥11 indicating clinically significant anxiety,^29^ while depressive symptoms were evaluated using the PHQ‐9.

Sleep pathology was quantified on POD 1–3 using the Athens insomnia scale (AIS),^30^, ^31^ with scores of 4–6 indicating sleep disturbances and scores > 6 indicating insomnia.

For manic manifestations, we applied the Young Mania Rating Scale (YMRS)^32^, ^33^ with a cutoff of 5, which has been empirically established to detect subclinical hypomania.^34^ Broad psychopathological features were captured using the 18‐item Brief Psychiatric Rating Scale (BPRS),^35^, ^36^ with scores > 30 indicating clinically significant impairment.^37^ Dissociative states were evaluated using the clinician‐administered dissociative states scale (CADSS),^38^, ^39^ with a score exceeding 0 indicating notable dissociation. These symptom assessments were conducted on POD 1–3, as well as 2 weeks and 1 month after surgery.

Psychological evaluation scale assessments, including the PHQ‐9, MADRS, YMRS, BPRS and CADSS, were performed at the patient's bedside before discharge from the hospital. Assessments at 2 weeks and 1 month after surgery were conducted through video teleconference. Based on previous studies concerning the impact of ketamine on patients with depression, the MADRS was explicitly employed to assess depressive symptoms during the first 3 days after esketamine administration. The evaluators were psychiatrists with more than 5 years of clinical experience who had undergone specialized training and were blinded to group allocations.

### 2.9 Adverse events and other perioperative metrics

Adverse events were monitored daily on POD 1–3. We systematically recorded the occurrence of four predefined complications: nausea, vomiting, increased secretion and dizziness.

We also evaluated the surgical site, number of operations at the same locus, duration of surgery, duration of anaesthesia, blood loss volume, urine output, fluid replenishment volume and vital signs, including BP, HR, SpO_2_ and BIS. The postoperative recovery period, extubation timing and pain numeric rating scale (NRS) evaluations were recorded 2 and 4 h after surgery, as well as on POD 1–3. Inflammatory markers, such as leucocyte count, C‐reactive protein (CRP) level, interleukin‐6 (IL‐6) concentration and erythrocyte sedimentation rate (ESR), were also measured.

### 2.10 Sample size

The sample size calculation was explicitly based on the primary outcome measure (response rate). According to a previous study,^40^ the response rate for ketamine in patients with moderate‐to‐severe depression ranged from approximately 26% to 56%. The remission rate for anxiety and depression in the experimental group was approximately 30%.^41^ Given the clinical manifestations, a significant disparity of at least 20% was observed. We hypothesized response rates of 30% for the experimental (esketamine) group and 10% for the control group. A concurrent group design with a ratio of 1:1 was adopted, with an alpha level set at 0.05 and a power of 80%. The calculated sample size was 59 participants per group. To account for an expected 10% dropout rate, we planned to recruit 65 participants per group, giving a total of 130 participants.

### 2.11 Statistical analysis

Statistical analyses were performed using SPSS (version 22.0; IBM) and R software (version 4.4.2; R Foundation for Statistical Computing). The normality of the continuous data distributions was assessed using the Kolmogorov–Smirnov test. Continuous variables were described using the mean ± standard deviation for normally distributed data and compared between groups using the independent samples t‐test. The median and interquartile range (IQR) were used for data that did not conform to a normal distribution, and intergroup differences were assessed using the Mann–Whitney *U* test. Categorical variables were reported as frequencies and percentages, with intergroup comparisons performed using the chi‐square test or Fisher's exact test, as appropriate. For categorical outcomes, relative risk (RR) and 95% confidence intervals (CI) were calculated. All between‐group comparisons were performed separately at each time point without cross‐temporal comparisons. To account for within‐subject correlation, MADRS scores were analysed as a continuous variable using mixed‐effects models with random intercepts for each participant to estimate the overall treatment effect across time points, with results presented as adjusted mean differences (aMD) and 95% CI. All statistical tests were two‐tailed, and a *p*‐value of less than .05 was considered statistically significant. For multiple comparisons across time points or outcome measures, *p*‐values were adjusted using the Holm–Bonferroni method to control the family‐wise error rate, and expressed as adjusted *p*‐value (adj. *p*).

## 3 RESULTS

**FIGURE 1:**
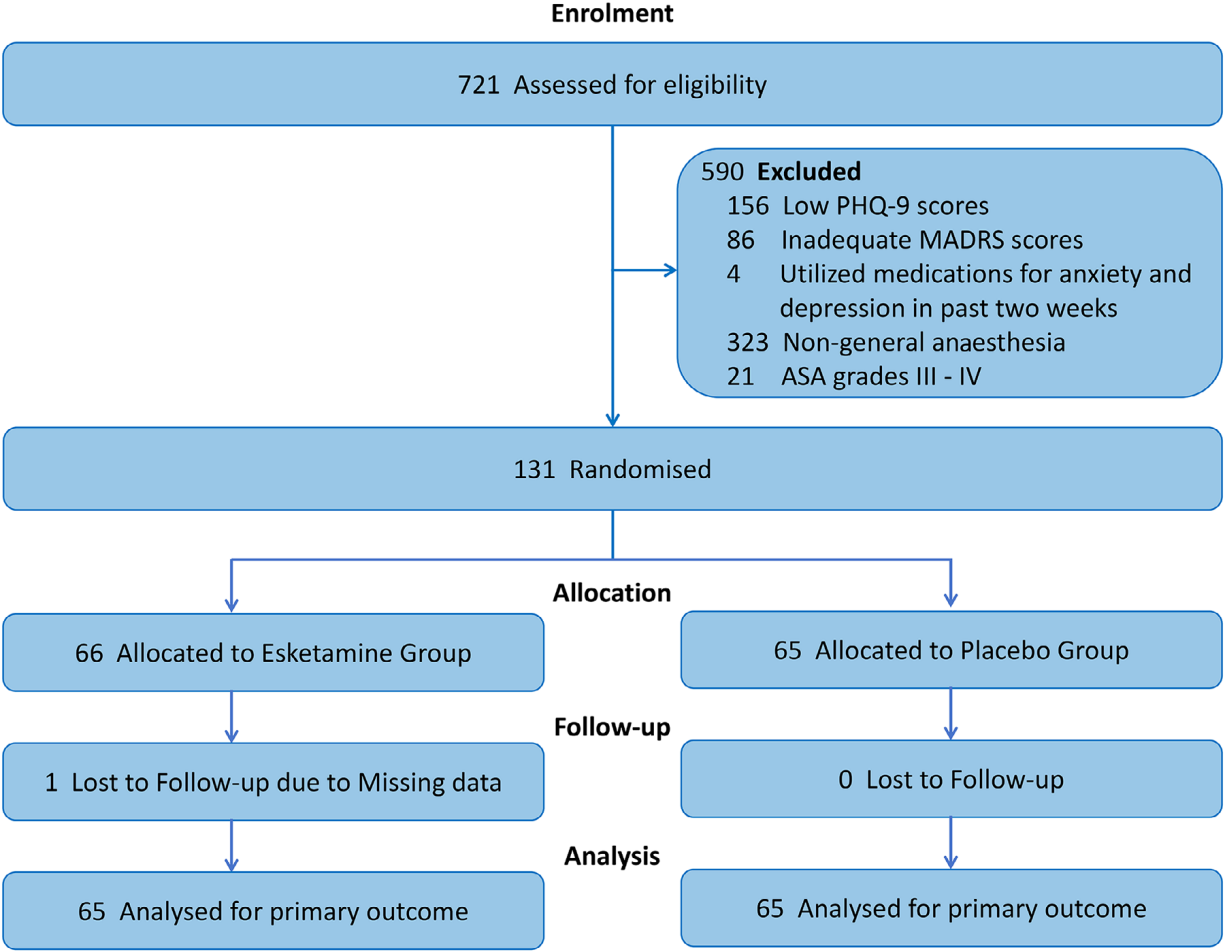
CONSORT flow diagram of the study.

This study initially screened 721 patients who underwent repeated surgical debridement, and a total of 131 subjects who met the analysis criteria and completed the randomized blinding procedure were included. Most patients were excluded for reasons such as a low PHQ‐9 score, inadequate MADRS score, lack of general anaesthesia, high ASA grade, or recent use of psychotropic medications. The enrolled patients were randomly assigned to the esketamine (*n* = 66) or placebo (*n* = 65) groups. One patient in the esketamine group was excluded due to data loss resulting from discharge on the first postoperative day, resulting in a final analysis cohort of 130 patients. A flowchart of patient enrolment and progression in this study is presented in Figure 1. The baseline characteristics and intraoperative details were well‐balanced between the two groups. Shapiro–Wilk tests showed that the number of surgeries, surgery duration, anaesthesia duration, total intra‐operative drug consumption, MADRS score, PHQ‐9 score and MMSE score were not normally distributed; these variables are therefore presented as median(IQR) in Table 1.

**TABLE 1:** Comparison of general characteristics between the two groups.

Characteristic	Esketamine group (*n* = 65)	Placebo group (*n *= 65)	*p* value
Age, mean ± SD, years	54.29 ± 13.84	51.17 ± 15.86	0.234
Height, mean ± SD, cm	167.17 ± 7.67	168.11 ± 7.6	0.487
Sex			0.860
Male, *n* (%)	31 (47.7)	30 (46.2)	
Female, *n* (%)	34 (52.3)	35 (53.8)	
BMI, mean ± SD, kg/m^2^	25.04 ± 3.71	25.59 ± 4.65	0.454
Education			1.000
High school and above, *n* (%)	36 (55.4)	36 (55.4)	
Up to high school, *n* (%)	29 (44.6)	29 (44.6)	
Smoking history			0.260
Non‐smoker, *n* (%)	40 (61.5)	38 (58.5)	
Occasional smoker (less than 2 times per week), *n* (%)	7 (10.80)	5 (7.7)	
Former smoker (not in the last year), *n* (%)	14 (21.5)	11 (16.9)	
Current smoker, *n* (%)	4 (6.2)	11 (16.9)	
Alcohol use			0.832
No alcohol use, *n* (%)	37 (56.9)	39 (60.0)	
Occasional drinker (less than 2 times per week), *n* (%)	16 (24.6)	12 (18.5)	
Former drinker (not in the last year), *n* (%)	10 (15.4)	11 (16.9)	
Current drinker, *n* (%)	2 (3.1)	3 (4.6)	
Hypertension, *n* (%)	20 (30.8)	20 (30.8)	1.000
Diabetes, *n* (%)	15 (23.10)	19 (29.20)	0.425
Hyperlipidaemia, *n* (%)	4 (6.2)	5 (7.7)	0.730
ASA			0.083
I, *n* (%)	4 (6.2)	10 (15.4)	
II, *n* (%)	61 (93.8)	55 (84.6)	
Allergy history, *n* (%)	14 (21.5)	14 (21.5)	1.000
Chronic pain, *n* (%)	19 (29.2)	15 (23.1)	0.425
Surgical sites			1.000
Chest, *n* (%)	6 (9.20)	7 (10.80)	
Abdomen, *n* (%)	9 (13.80)	5 (7.70)	
Back, *n* (%)	3 (4.60)	1 (1.50)	
Limbs, *n* (%)	37 (56.92)	43 (66.15)	
Others, *n* (%)	10 (15.38)	9 (13.80)	
Number of surgery^†^, median (IQR)	3 (2, 3)	2 (2,4)	0.562
Surgical Wound Classification			0.622
Class I, *n* (%)	10 (15.4)	11 (16.9)	
Class II, *n* (%)	30 (46.2)	30 (46.2)	
Class III, *n* (%)	22 (33.8)	22 (33.8)	
Class IV, *n* (%)	3 (4.6)	2 (3.1)	
Surgery duration^†^, median (IQR), min	85 (56.5, 126.6)	78 (55, 124.5)	0.458
Anaesthetic process			
Anaesthesia duration^†^, median (IQR), min	120(89, 166)	105 (83.5, 161.5)	0.486
Application of active warming, *n* (%)	33 (50.8)	24(36.90)	0.112
Remifentanil^†^, median (IQR), mg	0.8 (0.46, 1)	0.7(0.4, 1)	0.369
Propofol^†^, median IQR), mg	200 (127, 290)	200 (130, 300)	0.937
Sufentanil^†^, median (IQR), µg	30 (25, 35)	30 (30, 35)	0.384
NRS score			
Resting > 3, *n* (%)	6 (9.20)	7 (10.80)	0.770
Exercise > 3, *n* (%)	9 (13.80)	9 (13.80)	1.000
MADRS score^†^, median (IQR)	25 (24, 27)	24 (24, 28)	0.475
PHQ‐9 score^†^, median (IQR)	16 (14, 17)	16 (14, 18)	0.447
HADS‐A score > 11, *n* (%)	51 (78.50)	50 (76.90)	.833
MMSE score^†^, median (IQR)	29 (28, 29)	29 (28, 29)	0.882
BPRS score > 30, *n* (%)	0 (0.00)	3 (4.60)	0.080
CADSS score≠0, *n* (%)	4 (6.20)	4 (6.20)	1.000
YMRS score≥5, *n* (%)	13 (20.00)	21 (32.30)	0.110
AIS score > 6, *n* (%)	63 (96.90)	60 (92.30)	0.244

*Note*: Variables are delineated in terms of quantity (%), median (interquartile range), and standard deviation.

Abbreviations: ASA, American Society of Anesthesiologists; BPRS, Brief Psychiatric Rating Scale (18 items); CADSS, Clinician‐Administered Dissociative States Scale (27 items); HADS, Hospital Anxiety and Depression Scale; MADRS, Montgomery‐Åsberg Depression Rating Scale; PHQ‐9, Patient Health Questionnaire‐9 items; YMRS, Young Mania Rating Scale (11 items); MMSE, Mini Mental State Examination; NRS, Numerical Rating Scale; HDS‐A, Hospital Anxiety and Depression Scale—Anxiety subscale; AIS, Athens Insomnia Scale.

† median [IQR] due to non‐normal distribution.

### 3.1 Primary and Secondary Outcomes

Patients in the esketamine group exhibited higher response rates (≥50% decrease in the MADRS score from baseline) on each postoperative day than those in the placebo group. On POD 1, the response rate was 53.8% in the esketamine group versus 26.2% in the placebo group, corresponding to an RR of 2.06 with a 95% CI of 1.29–3.28 (adj. *p *= 0.003). This trend continued on POD 2, whereby the esketamine group had a response rate of 60.0% compared to 40.0% in the placebo group, corresponding to an RR of 1.62 with a 95% CI of 1.12–2.36 (adj. *p *= .018). On POD 3, the esketamine group maintained a significantly higher response rate of 73.8% compared to 53.8% in the placebo group, corresponding to an RR of 1.37 with a 95% CI of 1.05–1.79 (adj. *p *= .018) (Table 2).

**TABLE 2:** The differences in primary and secondary outcomes between the two groups.

	Esketamine group (*n* = 65)	Placebo group (*n* = 65)	RR or MD (95% CI)	Adj. *p* value
MADRS Response rate^1^				
POD 1, *n* (%)	35 (53.80)	17 (26.20)	2.06 (1.29–3.28)	0.003
POD 2, *n* (%)	39 (60.00)	26 (40.00)	1.62(1.12–2.36)	0.018
POD 3, *n* (%)	48 (73.80)	35 (53.80)	1.37 (1.05–1.79)	0.018
MADRS Remission rate^2^				
POD 1, *n* (%)	22 (33.80)	7 (10.80)	3.14 (1.44–6.84)	0.004
POD 2, *n* (%)	26 (40.00)	15 (23.10)	1.73 (1.02–2.96)	0.038
POD 3, *n* (%)	37 (56.90)	15 (23.10)	2.47 (1.51–4.03)	<.001
PHQ‐9 score on POD 3, median (IQR)	6 (5, 8)	8 (6, 12)	2.09 (.76–3.43)	0.004
HADS‐A score ≥11 on POD 3, *n* (%)	6 (9.20)	15 (23.10)	0.40 (0.17–60.97)	0.032
Psychiatric outcomes				
YMRS score≥5				
POD 1, *n* (%)	4 (6.20)	3 (4.60)	1.33 (0.31–5.72)	0.930
POD 2, *n* (%)	3 (4.60)	1 (1.50)	3.00 (0.32–28.09)	0.930
POD 3, *n* (%)	3 (4.60)	1 (1.50)	3.00 (0.32–28.09)	0.930
2 weeks after surgery, *n* (%)	3 (4.60)	0 (0)	–	–
1 month after surgery, *n* (%)	2 (3.10)	0 (0)	–	–
BPRS score > 30				
POD 1, *n* (%)	3 (4.60)	4 (6.20)	0.75 (0.17–3.22)	1.000
POD 2, *n* (%)	1 (1.50)	4 (6.20)	0.25 (0.03–2.18)	0.684
POD 3, *n* (%)	1 (1.50)	4 (6.20)	0.25 (0.03–2.18)	0.684
2 weeks after surgery, *n* (%)	1 (1.50)	1 (1.50)	1.00 (0.06–15.65)	1.000
1 month after surgery, *n* (%)	0 (0)	3 (4.60)	–	–
CADSS score > 0				
POD 1, *n* (%)	1 (1.50)	2 (3.10)	0.50 (0.05–5.38)	1.000
POD 2, *n* (%)	1 (1.50)	1 (1.50)	1.00 (0.06–15.65)	1.000
POD 3, *n* (%)	3 (4.60)	1 (1.50)	3.00 (0.32–28.09)	0.930
2 weeks after surgery, *n* (%)	0 (0)	0 (0)	–	–
1 month after surgery, *n* (%)	0 (0)	0 (0)	–	–

*Note*: Response rate 1 was defined as a reduction of no less than 50% in the baseline MADRS score; Remission rate 2 was defined as a MADRS score not exceeding 10.

Abbreviations: MADRS, Montgomery–Asberg Depression Rating Scale; PHQ‐9, Patient Health Questionnaire‐9 items; HADS‐A, Hospital Anxiety and Depression Scale—Anxiety subscale; BPRS, Brief Psychiatric Rating Scale (18 items); YMRS, Young Mania Rating Scale (11 items); CADSS, Clinician‐Administered Dissociative States Scale (27 items); CI, Confidence Interval; Diff, Difference.

**FIGURE 2:**
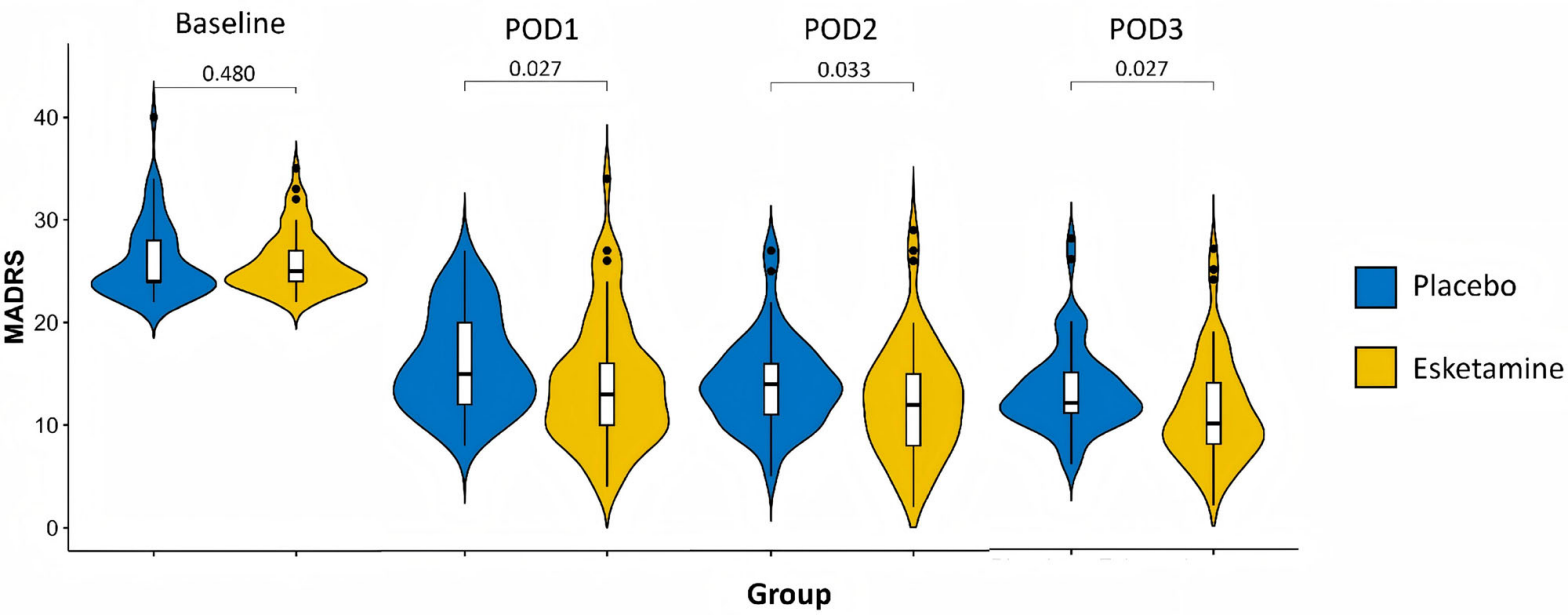
Comparison of MARDS between the two groups of patients at each time point.

For secondary outcomes, the remission rate, defined as MADRS ≤10, was significantly higher in the esketamine group on each POD. On POD‐1, 33.8% of patients achieved remission versus 10.8% in the placebo group (RR: 3.14, 95% CI: 1.44–6.84, adj. *p *= .004). On POD‐2, 40.0% achieved remission versus 23.1% (RR: 1.73, 95% CI: 1.02–2.96, adj. *p *= 0.038). By POD‐3, the esketamine group had a 56.9% remission rate compared to 23.1% in the placebo group (RR, 2.47; 95% CI, 1.51–4.03; adj. *p *< .001) (Table 2). Improvement in depressive symptoms, as assessed based on the MADRS score, was also significantly greater in the esketamine group at each postoperative time point (POD 1: aMD: ‐1.97, 95% CI: ‐3.505 to ‐0.433, adj. *p* = 0.027; POD 2: aMD: ‐1.68, 95% CI: ‐3.213 to ‐0.141, adj. *p* = 0.033; POD 3: aMD: ‐2.046, 95% CI: ‐3.582 to ‐0.510, adj. *p* = 0.027) (Figure 2). The psychiatric adverse effects of esketamine, including manic symptoms, dissociative symptoms and other psychotomimetic effects (YMRS, CADSS and BPRS), were also assessed at multiple time points (3 days, 2 weeks and 1 month after surgery). No statistically significant differences were observed between the two groups at any of the time points (Table 2).

Furthermore, patients in the esketamine group had significantly lower PHQ‐9 scores on POD 3, indicating significantly reduced depressive symptoms (6 vs. 8, *p* = .004). The proportion of patients with anxiety (HADS‐A) was also lower in the esketamine group on POD 3 (9.2% vs. 23.1%, *p* = .032) (Table 2).

### 3.2 Adverse Events

Within the 3‐day postoperative period, adverse events such as nausea, vomiting, dizziness and increased secretions were not significantly different between the esketamine and placebo groups. On POD 1, nausea was reported in 12 (18.5%) versus 17 (26.2%) participants and vomiting in 11 (16.9%) versus 15 (23.1%) participants in the esketamine and placebo groups, respectively. On POD 2, only the placebo group recorded three new cases of nausea (4.6%) and one case of vomiting (1.5%), whereas no esketamine‐treated patient experienced these events. Dizziness on POD 1 occurred equally in both groups (7 cases, 10.8% each), and no further episodes of dizziness or increased secretions were observed on POD 2 or POD 3 (Table 3). Similarly, vital signs remained stable across all recorded time points, with no significant differences between the groups (Tables S1–S4). Postoperatively, there were no significant differences in inflammatory markers between the two patient groups (Table S5). Additionally, there was no conspicuous discrepancy in the pain scores following rest and exercise between the two groups at 2 and 4 h after surgery or on POD 1–3 (Table S6). The two groups showed no statistically significant differences in terms of sleep quality (Table S7). For further details, please refer to Tables S1–S7.

**TABLE 3:** Adverse events occurring within the first three postoperative days between the two groups.

	Esketamine group (*n* = 65)	Placebo group (*n* = 65)	Adj. *p* value
POD 1			
Nausea, *n* (%)	12 (18.50)	17 (26.20)	0.876
Vomiting, *n* (%)	11 (16.90)	15 (23.10)	0.876
Increased secretions, *n* (%)	0 (0.0)	0 (0.0)	—
Dizziness, *n* (%)	7 (10.80)	7 (10.80)	1.000
POD 2			
Nausea, *n* (%)	0 (0.0)	3 (4.60)	0.160
Vomiting, *n* (%)	0 (0.0)	1 (1.50)	0.315
Increased secretions, *n* (%)	0 (0.0)	0 (0.0)	—
Dizziness, *n* (%)	0 (0.0)	0 (0.0)	—
POD 3	0 (0.0)	0 (0.0)	
Nausea, *n* (%)	0 (0.0)	0 (0.0)	—
Vomiting, *n* (%)	0 (0.0)	0 (0.0)	—
Increased secretions, *n* (%)	0 (0.0)	0 (0.0)	—
Dizziness, *n* (%)	0 (0.0)	0 (0.0)	—

### 3.3 Subgroup Analysis

We conducted subgroup analyses stratified by age, sex and education level. In each subgroup, the MADRS scores tended to be lower in the esketamine group compared with the control group; however, none of these differences remained statistically significant after adjustment for multiple comparisons, likely due to insufficient sample sizes within subgroups. The non‐significant between‐group *p*‐values across all subgroups suggest that the treatment effects were consistent across different demographic characteristics, indicating robustness of the findings across diverse patient populations (Table S8).

## 4 DISCUSSION

This study employed a randomized, double‐blind, placebo‐controlled design to investigate the effect of a single intraoperative administration of subanaesthetic‐dose esketamine (.2–.3 mg/kg) on postoperative emotional outcomes in patients with moderate‐to‐severe anxiety or depression requiring repeated debridement surgeries. The results indicated that, compared with the placebo group, esketamine significantly improved postoperative anxiety and depressive symptoms, with an approximately 20% higher remission rate at three days post‐surgery. Our findings are consistent with and extend the existing evidence on the utility of low‐dose esketamine in the perioperative setting. Previous studies indicate that subanaesthetic doses not only achieve a plateau in antidepressant effects^42^ but also exert minimal impact on circulatory, respiratory,^43^ and neuropsychiatric functions while reducing the risk of paradoxical tolerance associated with excessive NMDA receptor blockade.^44^The present study corroborates this favourable profile and further demonstrates its efficacy in the specific, psychologically vulnerable population of patients undergoing multiple wound surgeries.

The rapid alleviation of depressive and anxiety symptoms observed in this study may be closely related to the unique mechanism of action of esketamine. Unlike conventional general anaesthetic agents (e.g., propofol, inhaled anaesthetics and benzodiazepines), which primarily exert their effects by enhancing gamma‐aminobutyric acid (GABA)‐ergic inhibition, leading to widespread cortical suppression and sedation, esketamine functions as a non‐competitive N‐methyl‐D‐aspartate (NMDA) receptor antagonist. Its core mechanism lies in the blockade of the excitatory glutamatergic system.^44^ Of particular interest is the hypothesis that the dissociative state induced by NMDA receptor antagonism may create a psychological ‘reset’ window for patients. This transient alteration in consciousness can temporarily disrupt the persistent cycle of negative rumination associated with their underlying condition or surgical trauma.^45^, ^46^ This mechanistic distinction may explain why esketamine has a potential advantage over traditional sedative agents in rapidly improving perioperative mood.

This study found that a single intraoperative subanaesthetic dose of esketamine rapidly improved postoperative anxiety and depression in patients undergoing multiple surgeries. This finding is consistent with the conclusion of a recent review encompassing 67 trials,^47^ which confirmed the definite short‐ and long‐term efficacy of esketamine in preventing postoperative depression. More importantly, our study successfully extends this evidence to a high‐risk population with pre‐existing moderate‐to‐severe mood disorders who require multiple surgeries, providing direct evidence for the application of this drug in this specific clinical scenario. A previous meta‐analysis of 11 randomized controlled trials indicated that the prophylactic use of esketamine could significantly alleviate postoperative depressive symptoms (SMD: ‐0.61; 95% CI: ‐0.96 to ‐0.25; *p* = 0.0008).^48^ This conclusion aligns with and reinforces the primary findings of the present study, collectively supporting the positive role of esketamine in perioperative mood management. However, it is important to note that the patient population included in that meta‐analysis differs significantly from the target population of this study; it did not specifically focus on patients who required multiple surgeries and presented with pre‐existing moderate‐to‐severe anxiety and depression. Previous studies on esketamine have reported inconsistent findings. Such as the study by Lii et al.,^49^ which did not observe a significant antidepressant effect of perioperative ketamine. A direct comparison is limited by fundamental differences in trial design, particularly regarding the enrolled patient population and sample size. Our study specifically focused on patients with preoperative, symptomatic distress in the context of repeated surgeries, whereas the aforementioned trial primarily included patients with diagnosed major depressive disorder. Furthermore, our larger sample size may have provided greater statistical power to detect a clinically meaningful effect in this targeted population. Qiu et al.^15^ reported a significant reduction in postoperative depression with esketamine (0.3 mg/kg/h) in patients undergoing gynaecological laparoscopic surgery (OR 1.31, 95% CI 1.01–1.70), while no significant difference was observed in anxiety scores, which is inconsistent with the findings of the present trial. This discrepancy may stem from differences in the patient populations studied; their study involved patients without multiple surgeries, whereas our trial specifically focused on individuals who had undergone multiple surgical procedures. Luo et al. noted some alleviation of postoperative anxiety and depression with a single 0.5 mg/kg injection of esketamine during non‐cardiothoracic surgeries,^17^ and there is a discrepancy in the effective dose of esketamine between that trial and the present study. Although improvements in postoperative anxiety and depression were observed at both dose levels, Luo's trial did not document psychotomimetic or other neuropsychiatric adverse events. However, the safety profile of this dosing regimen remains incompletely characterized. Liu et al. conducted a study administered 0.25 mg/kg of esketamine intraoperatively in elective caesarean section surgeries, and then continued administration via patient‐controlled intravenous analgesia (PCIA) to evaluate the effect on postpartum depressive symptoms. Over the 42‐day follow‐up, anxiety and depression scores did not differ significantly between the esketamine and control groups, indicating that this low‐dose regimen did not reduce the incidence of postpartum mood disorders.^50^ The administration methods of esketamine and target populations differed from those in the present study, which may account for discrepancies between outcomes. Variability in these outcomes may be attributed to differences in patient populations, severity of preoperative anxiety and depression, scoring scales, drug administration methods, or timing of outcome assessments.

In our trial, we used a subanaesthetic dose of esketamine, which was lower than the typical anaesthetic dose and was administered for 20 min. Wang et al.^51^ reported that the use of subanaesthetic doses of esketamine might carry a higher risk of transient psychiatric symptoms; however, no similar adverse reactions were observed in our study. This discrepancy may be attributed to the combined use of propofol in our anaesthesia protocol, which may have reduced the risk of postoperative psychiatric symptoms.^52^ Vital signs remained stable throughout the procedure, and there were no significant differences in postoperative pain scores, as measured using the NRS, suggesting that the observed effects on anxiety and depression were not influenced by postoperative pain. The safety profile of a single intraoperative dose of esketamine was favourable, with only mild and similar adverse events observed in the two groups at all postoperative time points.

Despite the highly encouraging findings, our study has some limitations that should be considered when interpreting the results. Firstly, to ensure the rigor of the study design, this research limited the population to patients undergoing general anaesthesia who presented with moderate‐to‐severe anxiety and depression identified through preoperative screening, but without a prior formal diagnosis of depressive disorder. While this strategy significantly enhanced internal validity and ensured the reliability of the core findings, it may, to some extent, affect the generalizability of the results to broader clinical scenarios, such as regional anaesthesia or patients with stable depression. Secondly, the present study is limited by a relatively short observation period and the absence of preoperative psychological baseline data. Consequently, we are unable to assess the long‐term efficacy of esketamine or definitively ascertain the causal relationship between the observed symptoms and the patients' history of prior surgeries. Future investigations should incorporate earlier assessments, longer‐term follow‐up, and systematic monitoring of suicide risk to comprehensively elucidate emotional trajectories and evaluate the safety profile of sustained dosing regimens. Thirdly, the administration of esketamine in this study was integrated into the general anaesthesia protocol. Given that patients remained sedated or under general anaesthesia for at least 1 hour after drug administration, we were unable to conduct psychiatric rating scale assessments during this period. This resulted in the most significant dissociative and psychiatric symptoms occurring within the first hour post‐infusion not being fully captured, which may have led to a potential underestimation of the true incidence of acute psychiatric adverse events associated with esketamine. In view of these limitations, further research is needed to ensure that the benefits of such findings can be extended to a broader patient population.

## 5 CONCLUSIONS

Our findings indicate that intraoperative administration of esketamine can rapidly alleviate moderate‐to‐severe anxiety and depressive symptoms within 3 days after surgery in patients undergoing multiple surgical procedures, suggesting a beneficial role in mitigating acute perioperative emotional distress. The safety profile of esketamine remained favourable within 30 days postoperatively, with no observed increase in adverse events. It should be noted, however, that these symptomatic improvements do not necessarily equate to a sustained antidepressant effect. Future studies are warranted to further validate the efficacy and safety of esketamine in the perioperative management of moderate‐to‐severe anxiety and depression through expanded sample sizes and extended follow‐up durations.

## AUTHOR CONTRIBUTIONS

**Xiaomeng Yu**: Conceptualization; methodology; software; validation; project administration; formal analysis; investigation; data curation; writing—original draft; writing—review and editing. **Tianqi Shen**: Software; formal analysis; visualization; writing—original draft. **Ting Zhang**: Methodology; software; investigation; writing—original draft. **Rui Bao, Ziyi Guo, Ziyi Feng**: Investigation; data curation; writing—original draft. **Xiaoying Zhang, Li Tong**: Methodology; validation; formal analysis; writing—review and editing. **Guanyong Sun**: Conceptualization; writing—review and editing. **Weidong Mi**: conceptualization; writing—review and editing. **Jingsheng Lou**: Conceptualization; methodology; software; writing—review and editing. **Qiang Fu**: Conceptualization; methodology; writing—review and editing.

## CONFLICT OF INTEREST STATEMENT

The authors declare no conflicts of interest.

## CONSENT STATEMENT

Written informed consent was obtained from all patients for participation in the study as well as for the publication of this study and other reports.

## Supporting information



Supporting Information

## Data Availability

We sincerely hope that this rigorously de‐identified dataset will serve as a valuable shared resource for the research community, fostering deeper exploration and novel discoveries in diverse fields. Interested investigators are warmly invited to contact the corresponding author with a scientifically sound rationale to obtain de‐identified data and contribute collectively to scientific advancement.
