# Supplementary material for: Esketamine hydrochloride in the management of moderate‐to‐severe depressive symptoms in patients undergoing multiple wound repair surgeries: A multi‐centre randomized, double‐blind, placebo‐controlled trial
Source: Clin Transl Med. 2026 Mar 20;16(3):e70641. doi: 10.1002/ctm2.70641 (PMC13093759; doi:10.1002/ctm2.70641)
Supplement: Supplementary file 1 — Supporting Information [file CTM2-16-e70641-s001.docx]

**Supplementary Table**

Vital Signs Chart at Different Time Points During Surgery

| Table S1.SBP at each time point. | | | |
| --- | --- | --- | --- |
| Time points | Esketamine Group (n=65) | Placebo group (n=65) | *P* value |
| T_0_, mean ± SD, mmHg | 140.12±17.54 | 138.72±17.23 | 0.647 |
| T_1_, mean ± SD, mmHg | 127.25±21.17 | 125.11±18.41 | 0.540 |
| T_2_, median(IQR), mmHg | 127.00(116.00,142.50) | 127.00(110.50,146.00) | 0.633 |
| T_3_, median(IQR), mmHg | 106.00(100.00,113.50) | 105.00(98.00,117.50) | 0.430 |
| T_4_, median(IQR), mmHg | 111.00(104.00,126.00) | 116.00(104.00,126.00) | 0.785 |
| T_5_, median(IQR), mmHg | 106.00(98.50,120.00) | 108.00(102.00,118.00) | 0.335 |
| T_6_, mean ± SD, mmHg | 141.00±19.99 | 137.98±17.28 | 0.262 |
| T_0_, baseline; T_1_, after induction;T_2_, immediately after intubation; T_3_, 5 minutes after intubation; T_4_, at the beginning of the surgery; T_5_, at the end of the surgery; T_6_, at the time of awakening | | | |

| Table S2. DBP at each time point. | | | |
| --- | --- | --- | --- |
| Time points | Esketamine Group (n=65) | Placebo group (n=65) | *P* value |
| T_0_, mean ± SD, mmHg | 84.48±11.68 | 82.55±11.03 | 0.337 |
| T_1_, mean ± SD, mmHg | 79.66±12.07 | 79.75±14.36 | 0.968 |
| T_2_, mean ± SD, mmHg | 81.02±12.28 | 79.15±14.12 | 0.424 |
| T_3_, mean ± SD, mmHg | 72.25±10.85 | 72.86±11.72 | 0.757 |
| T_4_, median(IQR), mmHg | 68.00(63.00,75.50) | 73.00(67.00,80.00) | 0.373 |
| T_5_, median(IQR), mmHg | 72.00(67.00,81.00) | 72.00(66.00,86.00) | 0.941 |
| T_6_, median(IQR), mmHg | 94.00(85.00,98.00) | 91.00(83.00,97.00) | 0.192 |
| T_0_, baseline; T_1_, after induction; T_2_, immediately after intubation; T_3_, 5 minutes after intubation; T_4_, at the beginning of the surgery; T_5_, at the end of the surgery; T_6_, at the time of awakening | | | |

| Table S3. Heart rate at each time point. | | | |
| --- | --- | --- | --- |
| Time points | Esketamine Group (n=65) | Placebo group (n=65) | *P* value |
| T_0_, mean ± SD, beats/min | 78.42±12.05 | 79.11±16.00 | 0.781 |
| T_1_, mean ± SD, beats/min | 76.20±12.72 | 76.45±12.64 | 0.912 |
| T_2_, mean ± SD, beats/min | 80.98±14.70 | 79.88±15.16 | 0.673 |
| T_3_, mean ± SD, beats/min | 71.51±12.06 | 72.95±14.45 | 0.537 |
| T_4_, median(IQR), beats/min | 61.00(57.00,68.00) | 62.00(55.00,72.50) | 0.950 |
| T_5_, median(IQR), beats/min | 64.00(57.00,70.00) | 62.00(56.00,67.00) | 0.334 |
| T_6_, mean ± SD, beats/min | 83.31±12.38 | 85.15±4.59 | 0.438 |
| T_0_, baseline; T_1_, after induction; T_2_, immediately after intubation; T_3_, 5 minutes after intubation; T_4_, at the beginning of the surgery; T_5_, at the end of the surgery; T_6_, at the time of awakening | | | |

| Table S4. BIS at each time point. | | | |
| --- | --- | --- | --- |
| Time points | Esketamine Group (n=65) | Placebo group (n=65) | *P* value |
| T_0_, median(IQR) | 95(94,96) | 95(94,96) | 0.716 |
| T_1_, median(IQR) | 43(42,46) | 45(43,46) | 0.341 |
| T_2_, median(IQR) | 46(45,50) | 46(45,50) | 0.628 |
| T_3_, median(IQR) | 50(47,52) | 49(46,51) | 0.260 |
| T_4_, median(IQR) | 50(47,51) | 48(47,51) | 0.559 |
| T_5_, median(IQR) | 63(56,71) | 60(57,69) | 0.408 |
| T_6_, median(IQR) | 97(95,97) | 96(95,97) | 0.135 |
| T_0：_baseline; T_1_, after induction; T_2_, immediately after intubation; T_3_, 5 minutes after intubation; T_4_, at the beginning of the surgery; T_5_, at the end of the surgery; T_6_, at the time of awakening | | | |

| Table S5. Differences in Postoperative Inflammatory Markers Between Two Groups | | | |
| --- | --- | --- | --- |
|  | Esketamine Group (n=65) | Placebo group (n=65) | *P* value |
| WBC, mean ± SD, ×10^9^/L | 6.46±2.01 | 7.00±2.19 | 0.145 |
| IL-6, median(IQR), pg/mL | 5.78(2.29,11.02) | 6.72(2.01,11.47) | 0.966 |
| CRP, median(IQR), mg/L | 0.45(0.19,2.20) | 0.79(0.28,2.20) | 0.315 |
| HBP, median(IQR), ng/mL | 45.74(31.52,67.28) | 50.61(34.69,69.71) | 0.401 |
| ESR, median(IQR), mm/h | 9.00(6.00,23.00) | 13.00(5.00,29.00) | 0.911 |
| WBC, white blood count; IL-6, interleukin-6; CRP, C-reactive protein; HBP, heparin-binding protein; ESR, erythrocyte sedimentation rate | | | |

| Table S6. Comparison of Numerical Rating Scale Scores at Various Postoperative Time Points Between Two Groups | | | |
| --- | --- | --- | --- |
| Indicators | Esketamine Group (n=65) | Placebo group (n=65) | *P* value |
| 2h |  |  |  |
| Rest-induced | 2(0,2) | 1(0,3) | 0.469 |
| Movement-induced | 2(1,3) | 2(1,4) | 0.913 |
| 4h |  |  |  |
| Rest-induced | 1(0,2) | 1(0,2) | 0.718 |
| Movement-induced | 2(0,3) | 2(1,3) | 0.977 |
| POD-1 |  |  |  |
| Rest-induced | 1(0,2) | 1(0,3) | 0.804 |
| Movement-induced | 2(2,4) | 2(1,5) | 0.801 |
| POD-2 |  |  |  |
| Rest-induced | 1(0,1) | 1(1,2) | 0.427 |
| Movement-induced | 2(1,3) | 2(1,4) | 0.278 |
| POD-3 |  |  |  |
| Rest-induced | 1(0,1) | 0(0,1) | 0.804 |
| Movement-induced | 1(1,2) | 1(1,2) | 0.428 |
| Table S6. Comparison of Numerical Rating Scale Scores at Various Postoperative Time Points Between Two Groups | | | |

Postoperative Pain Score

| Table S7. Comparison of Athens Insomnia Scale Scores at Various Postoperative Time Points Between Two Groups. | | | |
| --- | --- | --- | --- |
| Time points | Esketamine Group (n=65) | Placebo group (n=65) | *P* value |
| POD-1 | 8(6,12) | 9(7,13) | 0.447 |
| POD-2 | 8(5,11) | 8(6,11) | 0.607 |
| POD-3 | 8(4,8) | 8(4,9) | 0.735 |
|  | | | |

Table S8. Subgroup analysis of MADRS scores using mixed effect models

| Subgroups | No. of patients | POD 1 | | | POD 2 | | | POD 3 | | |
| --- | --- | --- | --- | --- | --- | --- | --- | --- | --- | --- |
|  |  | aMD (95% CI) | Adj. p-value | p-value for interaction | aMD (95% CI) | Adj. p-value | p-value for interaction | aMD (95% CI) | Adj. p-value | p-value for interaction |
| Total | 130 | -1.969 (-3.505 to -0.433) | 0.027 |  | -1.677 (-3.213 to -0.141) | 0.033 |  | -2.046 (-3.582 to -0.510) | 0.027 |  |
| Sex |  |  |  | 0.688 |  |  | 0.994 |  |  | 0.968 |
| Male | 61 | -2.319 (-4.227 to -0.412) | 0.054 |  | -1.683 (-3.590 to 0.225) | 0.086 |  | -2.019 (-3.927 to -0.112) | 0.078 |  |
| Female | 69 | -1.687 (-4.048 to 0.674) | 0.326 |  | -1.671 (-4.032 to 0.690) | 0.326 |  | -2.083 (-4.444 to 0.278) | 0.255 |  |
| Age |  |  |  | 0.915 |  |  | 0.453 |  |  | 0.463 |
| <55 | 67 | -1.877 (-3.998 to 0.245) | 0.096 |  | -2.150 (-4.271 to -0.029) | 0.096 |  | -2.536 (-4.657 to -0.415) | 0.060 |  |
| ≥55 | 63 | -2.042 (-3.175 to 0.153) | 0.210 |  | -0.979 (-3.175 to 1.217) | 0.432 |  | -1.391 (-3.587 to 0.805) | 0.432 |  |
| Education |  |  |  | 0.667 |  |  | 0.590 |  |  | 0.936 |
| High school and above | 72 | -1.585 (-3.623 to 0.453) | 0.127 |  | -2.168 (-4.205 to -0.130) | 0.114 |  | -2.118 (-4.156 to -0.080) | 0.144 |  |
| Up to high school | 58 | -2.266 (-4.507 to -0.026) | 0.147 |  | -1.315 (-3.556 to 0.926) | 0.251 |  | -1.991 (-4.232 to 0.250) | 0.166 |  |
